# Glenohumeral Osteoarthritis: Disease Burden, Current Understanding, and Gaps in Knowledge and Treatment

**DOI:** 10.1177/23259671251339428

**Published:** 2025-05-19

**Authors:** Michael R. Davies, Volker Musahl, Brian Forsythe, Jacob G. Calcei, C. Benjamin Ma

**Affiliations:** † Department of Orthopaedic Surgery, University of California, San Francisco, 1500 Owens Street, San Francisco, California, USA; ‡ University of Pittsburgh Medical Center, Pittsburgh, Pennsylvania, USA; § Midwest Orthopaedics at Rush, Chicago, Illinois, USA; ‖ University Hospitals, Cleveland, Ohio, USA; Investigation performed at University of California, San Francisco, California, USA

**Keywords:** glenohumeral osteoarthritis, chondral defect, rotator cuff, shoulder instability

## Abstract

**Background:**

Glenohumeral osteoarthritis (GHOA) is a prevalent, costly problem among the aging population. Despite this, the underlying pathobiology and pathomechanics of the disease have not been fully elucidated, and treatment options remain limited to date.

**Purpose:**

The goal of the 2024 Arthritis Foundation (AF) and AOSSM GHOA Think Tank was to review the present body of knowledge regarding GHOA and identify opportunities for future inquiry to ultimately improve patient outcomes.

**Study Design:**

Narrative review.

**Methods:**

This narrative review stems from the evidence-based discussion of the 2024 AF AOSSM Think Tank. This meeting was composed of 4 sessions, on the topics of “Burden of Disease and Shoulder OA,”“Shoulder Conditions and the Development of Shoulder OA,”“Treatment Strategies for Patients With Shoulder OA,” and “Designing and Conducting Multicenter Trials.” Each of these sessions was further divided into 4 lectures pertaining to each topic. Speaker presentations were made available for subsequent review and incorporation of content into this review.

**Results:**

The epidemiology of GHOA is reviewed, as well as the basic science of arthritis, the roles of repetitive throwing, shoulder instability, and rotator cuff disease in GHOA, along with existing treatment approaches.

**Conclusion:**

Several unanswered questions are in these fields that warrant future research are highlighted.

## The Burden of Disease

Primary osteoarthritis (OA) affecting the glenohumeral joint (GHOA), is a growing problem among the aging population, with a radiographic prevalence of 16% to 20% among middle-aged and elderly adults.^42^ Shoulder pain is likewise prevalent, with 16% of adults experiencing disabling shoulder pain daily.^64^ Among patients with atraumatic shoulder pain, primary GHOA is the second most common diagnosis after rotator cuff (RC) disease.^93^ Certain demographic risk factors for primary (idiopathic) GHOA include advanced age, female sex, smoking, elevated body mass index (BMI), hypertension, and hyperlipidemia.^93,101^ GHOA may also develop secondary to chronic shoulder instability or repetitive overuse in the setting of athletic activities or occupation.^11,56^ Finally, untreated RC tears may eventually result in the development of cuff tear arthropathy (CTA), marked by a distinct wear pattern and progression of anterosuperior escape.^29,73,98^ The goal of the 2024 Arthritis Foundation (AF) AOSSM Think Tank was to review and synthesize the evidence related to the pathobiology, pathomechanics, and treatment options for GHOA, while identifying gaps in knowledge and areas for future research. The resulting discussion is presented below.

## Pathobiology of OA and Targeted Therapies

Joint injury leads to progressive molecular tissue damage and chronic inflammation. The inflammatory process of joint injury leading to chondral breakdown has been well described in the knee OA literature, where this process can be conceptually divided into inflammation of the joint lining, or synovitis, and chondral breakdown.^59-61^ Synovitis is associated with elevated expression of interleukin-1 (IL-1) and downregulation of the IL-1 receptor antagonist.^58^ Chondral breakdown is mediated by numerous proteases and is perpetuated through cellular signaling with inflammatory and immune cells, as well as osteoblasts, osteocytes, and osteoclasts within the subchondral bone. Joint inflammatory markers such as C-terminal cross-linked telopeptides of type II collagen increase after an acute insult to the joint and remain elevated for several weeks after a surgical intervention, before declining to preoperative levels.^41,61^ Thus, surgery frequently results in a secondary insult to an inflamed joint. Timing of surgery relative to the acute joint injury is therefore an important clinical consideration.^40^ Ultimately, these posttraumatic and postsurgical pathways that have been identified in the knee OA literature require future validation in studies of GHOA.

Chronic inflammation and the persistence of inflammatory markers long after injury or surgical intervention have been studied through clinical trials, many of which have focused on knee OA.^31,32^ After knee injuries, as many as one-third of patients display signs of chronic inflammation and persistent inflammatory markers.^43^ Within the shoulder, there may also be specific inflammatory markers, together marking an “inflammatype,” which have not yet been defined. Future research is necessary to identify biomarkers that may be predictive of subsequent development of GHOA.^111^

Certain key pathways mediating OA have been identified in the knee OA literature, most notably the canonical Wnt (“Wingless/Integrated”) signaling pathway. Its prolonged activation appears to have a detrimental effect on cartilage.^21^ Conversely, activation of the TGF-β (transforming growth factor beta) pathway has been shown to play an important role in healthy cartilage homeostasis.^21^ Inhibition of the Wnt pathway through the small molecular modulator Lorecivivint has shown some early promise in the treatment of knee OA, with patients showing improvement in the Western Ontario and McMaster Universities Osteoarthritis Index (WOMAC) pain and function subscores at 24 weeks after treatment in a phase 2 randomized controlled trial.^108^ Treatment with the angiopoietin-like 3-derivative LNA043, which inhibits the Wnt pathway by increasing expression of Wnt signaling inhibitors Dickkopf-related protein 1 and frizzled-related protein, was shown to induce expression of hyaline cartilage synthesis genes and inhibit OA progression–related genes over ≥21 days, in a phase 1 randomized controlled trial.^33^ Additional therapeutic targets for improved chondrogenic differentiation include the proteoglycan Agrin, which has been shown to recruit and promote differentiation of chondrogenic growth/differentiation factor 5 (GDF5)-lineage joint-resident progenitor cells through inhibition of the Wnt pathway (Table 1).

**Table 1:** Summary of Biomolecular Factors Involved in Osteoarthritis

Name	Abbreviation	Role in Osteoarthritis
Interleukin-1	IL-1	Cytokine elevated in the setting of synovitis; marker of inflammation associated with osteoarthritis
Interleukin-1 receptor antagonist	IL-1RA	Cytokine that binds to the IL-1 receptor and prevents IL-1 from binding, preventing signaling through this pathway
C-terminal cross-linked telopeptides of type II collagen	CTX-II	Biomarker that is associated with cartilage breakdown
Wingless/Integrated	Wnt	Signaling pathway involved in cartilage homeostasis; prolonged activation may have a detrimental effect on cartilage
Transforming growth factor beta	TGF-β	Signaling pathway involved in cartilage homeostasis; activation promotes cartilage maintenance
Dickkopf-related protein 1	DKK-1	Inhibitor of canonical Wnt signaling
Frizzled-related protein	FRB	Inhibitor of canonical Wnt signaling
Runt-related transcription factor	Runx	Family of transcription factors downstream of TGF-β signaling; Runx2 is associated with hyaline cartilage production
Core binding factor beta	CBFβ	Subunit of a core-binding transcription factor involved in chondrocyte and osteoblast differentiation

Runx (Runt-related) transcription factors are downstream targets of TGF-β signaling.^30^ Activation of Runx2 transcription to increase hyaline cartilage production is another therapeutic target under early investigation in the setting of OA treatment.^63^ Sprifermin is a recombinant human fibroblast growth factor–18 analog, which can bind to the fibroblast growth factor receptor 3 receptor and in turn activate Runx2 transcription.^39,63^ Preclinical trials of Sprifermin have demonstrated an increase in hyaline cartilage and extracellular matrix production with treatment. In a subgroup analysis of the FGF-18 Osteoarthritis Randomized Trial with Administration of Repeated Doses knee OA trial, Sprifermin treatment resulted in a dose-dependent improvement in WOMAC pain scores as well as increased cartilage thickness on magnetic resonance imaging (MRI) at 3 years after initiation of treatment.^39^ Further studies have demonstrated that the Runx partner core binding factor β (CBFβ) is pivotal in chondrocyte and osteoblast differentiation and is decreased in expression in aged mouse articular cartilage and in human OA cartilage.^19,20,110^ Kartogenenin is a small molecule that regulates the CBFβ-Runt-related transcription factor 1 (Runx1) transcriptional program and enhances the synthesis of cartilage matrix components including type II collagen and aggrecan.^48^ Further clinical trials are required to investigate these signaling pathways and to establish the long-term efficacy of these molecular approaches to treating glenohumeral joint OA.

## Scapular Morphology and GHOA

Dr Gilles Walch described 3 major variants of primary GHOA based on glenoid morphology: type A, marked by a well-centered humeral head with concentric erosion of the glenoid; type B, marked by posterior subluxation of the humeral head corresponding to eccentric wear of the glenoid posteriorly; and type C, marked by glenoid retroversion of >25°.^99^ Correlation of glenoid morphology with RC muscle atrophy measured on MRI demonstrated that B-type glenoids, excessive retroversion, and posterior humeral head subluxation are associated with imbalances in RC muscle volume.^18,38^ An increased infraspinatus to subscapularis ratio was likewise directly correlated with a B2 glenoid (defined by a biconcave, retroverted morphology), increased retroversion, and increased humeral head subluxation in a multicenter cohort.^1^ In addition to cuff muscle asymmetries, increasing posterior deltoid muscle area has been correlated with increased retroversion in B2 glenoids.^77^ In patients who have glenohumeral instability, the subscapularis to infraspinatus/teres minor muscle area ratio has also been correlated with the direction of shoulder dislocation.^44^

Whether these muscular imbalances are the primary cause of the pattern of GHOA wear or resultant effects of preexisting bony anomalies remains unknown.^106^ A recent study found that B2 glenoids display significantly greater premorbid retroversion, as defined by referencing the anterior glenoid/paleoglenoid, compared with nonarthritic shoulders.^55^ Acromial dysplasia may represent an additional contributing factor to arthritic wear patterns, as posterior rotation of the coracoacromial arch is associated with shoulder instability.^17,45^ Thus, we conclude that scapular morphology and the surrounding shoulder musculature may have a multifactorial effect on eccentric wear patterns in GHOA, with muscle imbalances, premorbid retroversion, and acromial dysplasia all potentially playing a role.

## GHOA in Throwing Athletes

Repetitive high-intensity overhead activities correlate with specific degenerative changes observed in the shoulders of elite athletes.^85^ Javelin throwers have been shown to have high rates of internal rotation loss, partial-thickness or full-thickness RC tears, superior migration of the humeral head, and degenerative changes within the glenohumeral joint that correlate in severity with the duration of the high-performance phase of their careers.^90^ Former collegiate and professional baseball players have demonstrated persistent shoulder joint pain in 28% of 117 athletes surveyed.^14^ The phases of pitching most associated with injury to the shoulder are the late cocking and deceleration phases.^26^ In the late cocking phase, internal impingement may occur between the posterosuperior glenoid labrum and the articular side of the posterosuperior RC and greater tuberosity.^100^ This process eventually leads to labral pathology and articular-sided RC tears. In certain cases, extensive peel-back of the labrum may result in a type VIII superior labrum from anterior to posterior tear.^15^ The deceleration phase of throwing places the greatest forces on the RC and capsule as the muscles contract eccentrically to reduce the velocity of the arm.^2^

Repetitive throwing has also been associated with insufficiency of the anterior capsuloligamentous structures of the glenohumeral joint.^35^ Previous studies have noted significant anterior translational forces sustained during the late cocking/early acceleration phases of throwing, in addition to an increase of ~18° in external rotation corresponding to a 30% increase in anterior capsular laxity due primarily to elongation of the anterior inferior glenohumeral ligament (IGHL).^68,91^ Glenohumeral internal rotation deficit and scapular dyskinesia may result from poor throwing mechanics and a lack of stretching, in addition to poor core strength.^35^

In summary, the literature associating overhead throwing with GHOA remains limited; however, the available evidence suggests a concerning link to early degenerative changes that may progress to GHOA in the long term.

## GHOA in the Unstable Shoulder

Acute bony and cartilaginous lesions resulting from glenohumeral dislocations may ultimately progress to GHOA, although the natural history of this process remains unclear. Habermeyer et al^36^ described a stepwise progression of labroligamentous injury with an increasing number of instability events. This injury cascade begins with a Bankart lesion, followed by an IGHL attachment rupture (double lesion/Perthes), which may then progress to an anterior labral periosteal sleeve avulsion (triple lesion [ALPSA]). The process culminates in both labral and capsular degeneration (quadruple lesion).^36^ Compared with isolated Bankart lesions, ALPSA lesions are associated with 3 times the number of instability events before surgical intervention and carry a higher surgical failure rate of 17.4% to 19.2% versus 7.4% for isolated Bankart tears.^79^ ALPSA lesions are also more likely to be associated with other intra-articular pathology, including cartilage lesions of the joint.^3^

Glenolabral articular disruption (GLAD) lesions are acute cartilage injuries of the glenoid resulting from traumatic instability, which if left untreated may place patients at high risk for progression to arthritis.^81^ GLAD lesions carry a significantly higher risk of failure of arthroscopic treatment than Bankart lesions alone, which may in part be due to their biomechanical effect on translation of the humeral head.^105^ The incidence of acute glenoid chondral lesions in anterior shoulder instability patients ranges from 36% to 57%, compared with a 72% incidence of an anterior labral tear, and a 46% incidence of Hill Sachs lesions.^75,87^ The Multicenter Orthopaedic Outcomes Network shoulder instability cohort revealed overall rates of bone or cartilage lesions of 18.4% in patients undergoing primary anterior stabilization, and 47.6% in patients undergoing revision surgery.^27^ Risk factors independently associated with the presence of bone and cartilage lesions included male sex, revision surgery, Black race, higher BMI, increasing patient age, and lower preoperative 36-Item Short Form Health Survey Physical Component Summary.^27^ Additional evidence has shown that the risk of failure of arthroscopic stabilization increases with time from first dislocation >6 months and male sex.^82^

Treatment options for acute bone and cartilage injuries sustained in the setting of glenohumeral instability range from nonoperative measures, including nonsteroidal anti-inflammatory drugs, physical therapy (PT), and injections, to operative treatment. Surgical treatment may include debridement of the lesion, advancement of the labrum over the lesion, microfracture, osteochondral autograft transfer system (OATS), osteochondral allograft transplantation (OCA), or autologous chondrocyte implantation (ACI). Microfracture has shown a long-term success rate of 67% to 75%.^89,102^ However, in a study of military patients who underwent microfracture and labral repair compared with patients without an osteochondral injury who underwent labral repair alone, there was a lower return to active duty of 54% in the microfracture group versus labral repair alone (94%). Additionally, the microfracture group had worse patient-reported outcomes (PROs), suggestive of the negative effect of an osteochondral injury in this population.^34^

A case series of 7 patients who underwent OATS for full-thickness cartilage lesions of the shoulder demonstrated improved Constant scores at a mean of 8.75 years after surgery, with healing and a congruent joint surface in all but 1 patient.^53^ A case series of 20 patients who underwent OCA to the humeral head demonstrated incorporation in 18 out of 20 grafts and a high satisfaction rate (9 of 9 patients) among those who underwent an isolated humeral OCA, with a low satisfaction rate in patients with bipolar lesions (2 of 11 patients satisfied).^86^ A case series of 4 patients who underwent ACI for humeral cartilage defects demonstrated an improvement in PROs at a mean follow-up of 41 months, with follow-up MRI demonstrating satisfactory defect coverage.^13^

In a study assessing the long-term development of GHOA after open stabilization surgery with 5- to 20- year follow-up, the onset of OA correlated with the number of instability events preoperatively.^76^ In a second study of the presence of glenohumeral osteoarthrosis in 570 shoulders after stabilization surgery, the overall rate of postoperative OA over the 6.5-year mean follow-up period was 19.7%. OA correlated with older age at the time of surgery, increased number of preoperative dislocations, and longer follow-up.^16^ Early intervention with arthroscopic stabilization in patients who sustained a first-time shoulder dislocation before age 25 has been shown to reduce the risk of secondary dislocation and to improve functional outcomes compared with nonoperative treatment over a 2-year follow-up period.^83^

Compared with anterior instability, outcomes after treatment of posterior instability with respect to the development of GHOA remain less certain. Multiple studies have found that while surgical intervention for posterior instability is effective at treating pain, it has also been correlated with progression of GHOA compared with nonoperative care.^62,96^ Posterior shoulder dislocation events carry a high risk of posterior bone loss development, with each instability event being responsible for up to 5% bone loss.^7^ Moderate posterior glenoid bone loss, defined as >13.5%, is associated with an 88% presence of pain at final follow-up and a 33% revision surgery rate.^107^ Surgical management of posterior instability is largely dependent on bone loss, with glenoid bony augmentation recommended in the primary setting with >20% bone loss, or in the revision setting with 10% to 20% bone loss.^25^

In conclusion, a growing body of evidence suggests that early surgical intervention for shoulder instability is likely warranted, as it may prevent subsequent dislocations, bone loss, and ultimately, GHOA. Although the connection between an isolated traumatic shoulder dislocation and GHOA remains unclear, recurrent dislocations resulting in progressive chondral injury and bone loss accelerate the process of posttraumatic GHOA.

## RC Tears and GHOA

Acromial morphology may play a role in determining which patients are at risk for RC tendon tears and which are at risk of developing primary GHOA. The critical shoulder angle, measured between the glenoid fossa and the lateral border of the acromion on a Grashey radiograph, is elevated in patients with degenerative RC tears and reduced in patients with primary GHOA.^94^ The acromial index is also elevated in the setting of cuff tears compared with GHOA, while the lateral acromial angle has been found to be reduced.^109^ A study exploring 3-dimensional modeling of scapular morphology determined that patients with cuff tears exhibited higher glenohumeral joint forces and a higher instability ratio in abduction, while patients with GHOA had a higher shear-to-compressive force ratio compared with RC tear patients.^78^ Taken together, these data suggest that acromial and scapular morphology may dictate distinct degenerative pathologies of the shoulder and that the predisposing anatomic factors for RC tears and primary GHOA are separate, despite the shared demographic features among patients with these conditions.

Both increased bulk of the posterosuperior cuff muscles and fatty infiltration of those same muscles have been independently correlated with increased retroversion of the glenoid and humeral head subluxation.^72^ This suggests that the RC may potentially have both a primary role and a compensatory response to the development of glenoid deformity.

Although the role of RC pathology in primary GHOA may be ambiguous, the effect of untreated, large-to-massive RC tears in the development of secondary GHOA, or CTA, is well-described.^29,73,98^ The superior migration of the humeral head that may occur with a massive RC tear was described as early as 1793, and the term “cuff tear arthropathy” was coined by Charles Neer.^73^ The pathogenesis of arthritis in CTA is thought to be a combination of mechanical factors and nutritional factors that ultimately lead to destruction of the chondral surfaces after anterosuperior escape of the humeral head, in addition to remodeling of the acromion and proximal humerus.^88^

Relative to the high prevalence of degenerative RC tears, the rate of CTA developing from these tears is much lower, estimated by Neer to occur in roughly 4% of all cuff tears.^84^ Similarly, not all patients with massive RC tears will progress to CTA. While it seems that patients with massive cuff tears who maintain their range of motion >90° in all planes may be at less risk for progression to CTA than those with pseudoparalysis, advanced muscle degeneration, or those who have failed an RC repair, it remains unknown which patients will progress to CTA based on the diagnosis of a massive RC tear alone.^49,70^

The clinical outcomes of primary GHOA on the treatment of RC tears has been studied, as has the effect of RC tears on shoulder arthroplasty. In patients with mild GHOA, defined as Samilson and Prieto grade 1, who underwent repair of large-to-massive RC tears, there was no change in failure rate, pain, or functional outcomes at 2 years after surgery.^46^ In a study utilizing the Shoulder Osteoarthritis Severity score to quantify global shoulder pathology as it relates to postoperative changes in American Shoulder and Elbow Surgeons (ASES) scores after RC repair, the authors found that worse overall intra-articular pathology correlated with decreased improvement in ASES scores after surgery.^22^ When assessing the effect of RC tears on patients undergoing reverse total shoulder arthroplasty (RTSA), patients who had primary GHOA with an intact RC had higher Constant scores at 2 years after surgery than patients with primary GHOA and cuff lesions, or patients with CTA who were undergoing RTSA.^74^ Taken together, these data suggest that outcomes after a joint preservation surgery such as RC repair are negatively affected by the presence of early GHOA, and likewise, outcomes after RTSA can be negatively affected by the presence of CTA, even though this remains among the most common indications for RTSA.

The optimization of RC muscle quality via the prevention of atrophy and fatty infiltration is a growing area of interest within the field, as poor muscle quality is linked to failed repairs, which may in turn progress to CTA.^6^ Certain pharmacologic agents, including the FDA-approved β3-receptor agonist mirabegron, have shown promise in small animal studies of RC tears by halting the progression of muscle fatty infiltration and fibrosis via beige and brown adipose differentiation of mesenchymal progenitor cells within muscle.^103^ Blood flow restriction (BFR), in combination with low-intensity exercise, has also been investigated as a therapeutic approach to increasing muscle strength around the shoulder, with mixed results.^12,50,57^ High-quality clinical trials of pharmacologic agents such as mirabegron and rehabilitation techniques such as BFR are needed to better assess their potential efficacy in the treatment of RC muscle degeneration and the downstream effects on GHOA.

## GHOA Treatment Options

### Physical Therapy

The physical impairments caused by GHOA can be divided into 4 treatment categories for PT: muscle strength, range of motion, movement impairment (as a factor of pain), and night pain (sleep disturbance). When treating a patient with PT for GHOA, it is important to modify activity based on the pain-monitoring model, ensuring that pain levels after PT return to preactivity levels by the morning after a therapy session.^95^ In patients with excessive pain in the setting of GHOA, modalities such as ice, heat, and high-intensity sensory transcutaneous electrical nerve stimulation can be applied for pain control as an adjunct to therapy. When performing stretching exercises for GHOA, it is important to respect bony deformity that is limiting range of motion, as attempting to push past these structural limitations will result in excessive pain and possible injury. Encouraging continued therapeutic exercise in the setting of painful OA is recommended, as it has been shown to aid in pain relief and is safe for articular cartilage.^10^ In spite of these recommendations, the evidence for PT in the treatment of primary GHOA remains insufficient, largely owing to a lack of high-quality clinical trials, although some remain ongoing.^52^ The effect of PT on GHOA therefore represents an understudied area that warrants further longitudinal observational studies to better identify which patients may best respond to nonoperative care.

Factors beyond the disease burden of GHOA may additionally play a role in patient outcomes, including mental health, resilience, and level of preoperative education provided to patients.^23,66,97^ In particular, patients with worse mental health status are more likely to have poor outcomes after shoulder arthroplasty.^47^ These findings highlight the need to provide feasible interventions to address mental health and resilience and to ensure adequate preoperative education in patients who are being treated for GHOA, whether with PT or surgery.

### Injections

Corticosteroid injections are a mainstay of nonoperative care for GHOA, although limited data exist regarding their efficacy. A prospective cohort study of 29 patients (30 shoulders) who underwent corticosteroid injections for GHOA and were followed over a 1-year period found that patients with more severe preinjection shoulder dysfunction observed greater symptomatic relief than patients with milder dysfunction. Improvements in pain and function were limited in duration to 4 months after injection.^67^ In the setting of shoulder arthroplasty, several recent studies have found an association between corticosteroid injections and increased risk of prosthetic joint infection, with risks of infection up to 9.0% in patients who underwent injections within 1 month of surgery.^5,92^

A systematic review of 1879 patients treated nonoperatively with a median 6-month follow-up reviewed the efficacy of other injectable therapies, including hyaluronic acid (HA), bone marrow aspirate concentrate (BMAC), leukocyte-rich and leukocyte-poor platelet rich plasma and autologous conditioned serum.^51^ Thirteen of the 19 studies analyzed studied HA injections, and the majority of studies reported a short-term improvement in visual analog scale scores, although one study found no difference between HA injection and placebo saline control.^51^

In a double-blind randomized controlled trial of leukocyte-poor platelet-rich plasma compared with HA injection for GHOA, there were no significant differences in PROs or functional scores between groups over the 12-month follow-up period. Both groups demonstrated some improvement in pain scores beginning at 1 to 2 months after injection.^54^ A pilot randomized controlled trial of 25 shoulders treated with BMAC injection versus cortisone for GHOA found that BMAC resulted in improved PROs compared with cortisone over the 12-month follow-up period, with the exception of the Western Ontario Osteoarthritis of the Shoulder index, which did not differ between groups.^28^ Overall, intra-articular injectable therapies represent viable short-term treatment options for symptoms of GHOA; however, further high-quality evidence is needed to evaluate their true efficacy.

### Arthroscopy

Arthroscopic debridement of the shoulder for GHOA is a treatment option that has been investigated primarily for younger patients with early GHOA, or those who otherwise are not candidates for shoulder arthroplasty but have failed nonoperative management. In a case series of 25 patients with a mean follow-up of 34 months who underwent glenohumeral joint debridement, loose body removal, partial synovectomy, and subacromial bursectomy, 8% had results that were deemed excellent, 72% had good results, and 20% had unsatisfactory results, with 83% of patients who preoperatively had severe stiffness demonstrating an improvement in motion postoperatively.^104^ More recently, Millett et al^69^ described the approach of Comprehensive Arthroscopic Management (CAM) as a way of arthroscopically addressing known pain generators in the arthritic shoulder. This approach involves debridement of labral tears and loose chondral flaps, loose body removal, and a complete capsular release, humeral head osteoplasty, and axillary nerve neurolysis.^4,69^ CAM has demonstrated a survivorship of 75.3% at 5 years and 63.2% at ≥10 years postoperatively, with humeral head flattening and severe joint incongruity identified as risk factors for failure.^4^

### Arthroplasty

Shoulder replacement remains the standard of care for painful end-stage GHOA that has failed nonoperative treatment. Over the past decade, shoulder arthroplasty rates have dramatically increased,^8,24^ with a 3-fold increase observed in RTSA, a 1.5-fold increase in anatomic total shoulder arthroplasty (TSA), and a decline in hemiarthroplasty.^37^

Large retrospective database studies have identified an increase in perioperative adverse events associated with RTSA compared with TSA that cannot be explained by patient demographics or comorbidities.^9^ Overall, surgeon volume >10 shoulder arthroplasties per year has been correlated with lower odds of revision surgery within a 2-year follow-up period.^9^ In the United States, approximately 75% of all shoulder arthroplasties are performed by surgeons who perform <10 TSAs or RTSAs per year.^9^ Certain strategies have been developed to control for surgeon experience with shoulder arthroplasty, including computed tomography–based planning, patient-specific guides, augmented reality assistance, and robot-assisted total shoulder systems. Observation of the effect of scapulothoracic orientation on postoperative patient outcomes may likewise allow for further optimization of component positioning based on patient posture and anatomy.^71^ Future studies are needed to assess both the short-term and the long-term efficacy of these recent strategies to standardize outcomes for shoulder arthroplasty.

The postoperative complication profiles of both TSA and RTSA have evolved with advancements in technology, particularly related to implant design and materials science. For TSA, the leading complications are RC failure, glenoid component loosening, and infection.^80^ For RTSA, the most common complications include acromial/scapular fracture, instability, and persistent pain.^80^ However, it should be noted that overall complication rates for each procedure have decreased substantially over the past 10 to 15 years.^65^

## Inspiration for Future Multicenter Studies

Based on the review and discussion of the evidence above at the 2024 AF AOSSM GHOA Think Tank, the following questions have been posited as critical knowledge gaps that warrant future multicenter cohort studies and clinical trials:

1. What is the role of novel small-molecule therapies and recently developed pharmacological agents in the treatment of GHOA?
2. What is the natural history of traumatic shoulder instability as it relates to the pathogenesis of GHOA?
3. What are the defining risk factors for patients with RC tears that lead predictably to secondary GHOA?
4. What is the efficacy of PT in the treatment of GHOA?
5. What factors account for the increase in higher perioperative morbidity or better clinical outcome with RTSA versus TSA?

In formulating these unsolved queries, we hope to spark future multicenter collaborations that may further our understanding of GHOA to improve treatment options and patient outcomes.

## References

[1] Aleem AW Chalmers PN Bechtold D Khan AZ Tashjian RZ Keener JD. Association between rotator cuff muscle size and glenoid deformity in primary glenohumeral osteoarthritis. J Bone Joint Surg Am. 2019;101(21):1912. 31567672 10.2106/JBJS.19.00086

[2] Andrews JR Alexander EJ. Rotator cuff injury in throwing and racquet sports. Sports Med Arthrosc Rev. 1995;3(1):30.

[3] Arner JW Cooper JD Elrick BP , et al. Outcomes of arthroscopic anterior labroligamentous periosteal sleeve avulsion lesions: a minimum 2-year follow-up. Am J Sports Med. 2022;50(6):1512-1519. 35416079 10.1177/03635465221090902

[4] Arner JW Elrick BP Nolte P-C Haber DB Horan MP Millett PJ. Survivorship and patient-reported outcomes after comprehensive arthroscopic management of glenohumeral osteoarthritis: minimum 10-year follow-up. Am J Sports Med. 2021;49(1):130-136. 33175562 10.1177/0363546520962756

[5] Baksh N Nadarajah V Connors KM , et al. Does preoperative corticosteroid injection increase the risk of periprosthetic joint infection after reverse shoulder arthroplasty? J Shoulder Elbow Surg. 2023;32(7):1459-1464. 36737032 10.1016/j.jse.2023.01.008

[6] Bedi A Bishop J Keener J , et al. Rotator cuff tears. Nat Rev Dis Primers. 2024;10(1):1-20. 38332156 10.1038/s41572-024-00492-3

[7] Bedrin MD Owens BD Slaven SE , et al. Prospective evaluation of posterior glenoid bone loss after first-time and recurrent posterior glenohumeral instability events. Am J Sports Med. 2022;50(11):3028-3035. 35983958 10.1177/03635465221115828

[8] Best MJ Aziz KT Wilckens JH McFarland EG Srikumaran U. Increasing incidence of primary reverse and anatomic total shoulder arthroplasty in the United States. J Shoulder Elbow Surg. 2021;30(5):1159-1166. 32858194 10.1016/j.jse.2020.08.010

[9] Best MJ Fedorka CJ Haas DA , et al. Higher surgeon volume is associated with a lower rate of subsequent revision procedures after total shoulder arthroplasty: a national analysis. Clin Orthop Relat Res. 2023;481(8):1572. 36853863 10.1097/CORR.0000000000002605 PMC10344546

[10] Bricca A Juhl CB Steultjens M Wirth W Roos EM. Impact of exercise on articular cartilage in people at risk of, or with established, knee osteoarthritis: a systematic review of randomised controlled trials. Br J Sports Med. 2019;53(15):940-947. 29934429 10.1136/bjsports-2017-098661

[11] Brophy RH Marx RG. Osteoarthritis following shoulder instability. Clin Sports Med. 2005;24(1):47-56. 15636776 10.1016/j.csm.2004.08.010

[12] Brumitt J Hutchison MK Kang D , et al. Rotator cuff strength is not augmented by blood flow restriction training. Phys Ther Sport. 2021;52:305-311. 34742029 10.1016/j.ptsp.2021.10.013

[13] Buchmann S Salzmann GM Glanzmann MC Wörtler K Vogt S Imhoff AB. Early clinical and structural results after autologous chondrocyte transplantation at the glenohumeral joint. J Shoulder Elbow Surg. 2012;21(9):1213-1221. 22047789 10.1016/j.jse.2011.07.030

[14] Bullock GS Nicholson KF Waterman BR , et al. Persistent joint pain and arm function in former baseball players. JSES Int. 2021;5(5):912-919. 34505105 10.1016/j.jseint.2021.05.001 PMC8411053

[15] Burkhart SS Morgan CD Kibler WB. The disabled throwing shoulder: spectrum of pathology part II: evaluation and treatment of SLAP lesions in throwers. Arthroscopy. 2003;19(5):531-539. 12724684 10.1053/jars.2003.50139

[16] Buscayret F Edwards TB Szabo I Adeleine P Coudane H Walch G. Glenohumeral arthrosis in anterior instability before and after surgical treatment: incidence and contributing factors. Am J Sports Med. 2004;32(5):1165-1172. 15262638 10.1177/0363546503262686

[17] Chalmers PN Beck L Miller M , et al. Acromial morphology is not associated with rotator cuff tearing or repair healing. J Shoulder Elbow Surg. 2020;29(11):2229-2239. 32417045 10.1016/j.jse.2019.12.035

[18] Chalmers PN Beck L Miller M , et al. Glenoid retroversion associates with asymmetric rotator cuff muscle atrophy in those with Walch B-type glenohumeral osteoarthritis. J Am Acad Orthop Surg. 2020;28(13):547. 31517880 10.5435/JAAOS-D-18-00830 PMC7064422

[19] Che X Jin X Park NR , et al. Cbfβ Is a novel modulator against osteoarthritis by maintaining articular cartilage homeostasis through TGF-β signaling. Cells. 2023;12(7):1064. 37048137 10.3390/cells12071064 PMC10093452

[20] Chen W Lu Y Zhang Y , et al. Cbfβ regulates Wnt/β-catenin, Hippo/Yap, and Tgfβ signaling pathways in articular cartilage homeostasis and protects from ACLT surgery-induced osteoarthritis. Elife. 2024;13:e95640. 10.7554/eLife.95640 PMC11132684 38805545

[21] Cherifi C Monteagudo S Lories RJ. Promising targets for therapy of osteoarthritis: a review on the Wnt and TGF-β signalling pathways. Ther Adv Musculoskel. 2021;13:1759720X211006959. 10.1177/1759720X211006959 PMC8053758 33948125

[22] Chi HM Davies MR Vijittrakarnrung C , et al. Association of preoperative shoulder osteoarthritis severity score with change in American Shoulder and Elbow Surgeons score at 2 years after rotator cuff repair. Orthop J Sports Med. 2024;12(7):23259671241257825. 10.1177/23259671241257825 PMC11295224 39100214

[23] Daher M Boufadel P Lopez R , et al. Beyond the joint: exploring the interplay between mental health and shoulder arthroplasty outcomes. J Orthop. 2024;52:1-5. 38404698 10.1016/j.jor.2024.02.005 PMC10881441

[24] Day JS Lau E Ong KL Williams GR Ramsey ML Kurtz SM. Prevalence and projections of total shoulder and elbow arthroplasty in the United States to 2015. J Shoulder Elbow Surg. 2010;19(8):1115-1120. 20554454 10.1016/j.jse.2010.02.009

[25] Dickens JF Hoyt BW Kilcoyne KG LeClere LE. Posterior glenoid bone loss and instability: an evidence-based approach to diagnosis and management. J Am Acad Orthop Surg. 2023;31(9):429. 36848487 10.5435/JAAOS-D-22-00060

[26] Dillman CJ Fleisig GS Andrews JR. Biomechanics of pitching with emphasis upon shoulder kinematics. J Orthop Sports Phys Ther. 1993;18(2):402-408. 8364594 10.2519/jospt.1993.18.2.402

[27] Duchman KR Hettrich CM Glass NA , et al. The incidence of glenohumeral bone and cartilage lesions at the time of anterior shoulder stabilization surgery: a comparison of patients undergoing primary and revision surgery. Am J Sports Med. 2018;46(10):2449-2456. 29985051 10.1177/0363546518781331

[28] Dwyer T Hoit G Lee A , et al. Injection of bone marrow aspirate for glenohumeral joint osteoarthritis: a pilot randomized control trial. Arthrosc Sports Med Rehabil. 2021;3(5):e1431-e1440. 10.1016/j.asmr.2021.07.005 PMC8527259 34712981

[29] Ecklund KJ Lee TQ Tibone J Gupta R. Rotator cuff tear arthropathy. J Am Acad Orthop Surg. 2007;15(6):340. 17548883 10.5435/00124635-200706000-00003

[30] Eldridge SE Barawi A Wang H , et al. Agrin induces long-term osteochondral regeneration by supporting repair morphogenesis. Sci Transl Med. 2020;12(559):eAAX9086. 10.1126/scitranslmed.aax9086 32878982

[31] Ericsson YB Roos EM Frobell RB. Lower extremity performance following ACL rehabilitation in the KANON-trial: impact of reconstruction and predictive value at 2 and 5 years. Br J Sports Med. 2013;47(15):980-985. 24029859 10.1136/bjsports-2013-092642

[32] Filbay SR Roos EM Frobell RB Roemer F Ranstam J Lohmander LS. Delaying ACL reconstruction and treating with exercise therapy alone may alter prognostic factors for 5-year outcome: an exploratory analysis of the KANON trial. Br J Sports Med. 2017;51(22):1622-1629. 28515057 10.1136/bjsports-2016-097124 PMC5754848

[33] Gerwin N Scotti C Halleux C , et al. Angiopoietin-like 3-derivative LNA043 for cartilage regeneration in osteoarthritis: a randomized phase 1 trial. Nat Med. 2022;28(12):2633-2645. 36456835 10.1038/s41591-022-02059-9 PMC9800282

[34] Green CK Scanaliato JP Sandler AB , et al. Simultaneous arthroscopic glenohumeral stabilization and glenoid microfracture in young, active-duty military patients: outcomes at 5-year follow-up. Orthop J Sports Med. 2023;11(2):23259671221146170. 10.1177/23259671221146170 PMC9900666 36756169

[35] Grossman MG Tibone JE McGarry MH , et al. A cadaveric model of the throwing shoulder: a possible etiology of superior labrum anterior-to-posterior lesions. J Bone Joint Surg Am. 2005;87(4):824. 15805213 10.2106/JBJS.D.01972

[36] Habermeyer P Gleyze P Rickert M. Evolution of lesions of the labrum-ligament complex in posttraumatic anterior shoulder instability: a prospective study. J Shoulder Elbow Surg. 1999;8(1):66-74. 10077800 10.1016/s1058-2746(99)90058-7

[37] Haddad DJ Rizvi OH Sherman NC Hamilton AR. Reverse and anatomic shoulder arthroplasty regional usage and open payment analysis using the Centers for Medicare and Medicaid Services database. J Shoulder Elbow Arthroplasty. 2024;8:24715492231207278. 10.1177/24715492231207278 PMC10860377 38348207

[38] Henninger HB Christensen GV Taylor CE , et al. The muscle cross-sectional area on MRI of the shoulder can predict muscle volume: an MRI study in cadavers. Clin Orthop Relat Res. 2020;478(4):871. 31725479 10.1097/CORR.0000000000001044 PMC7282568

[39] Hochberg MC Guermazi A Guehring H , et al. Effect of intra-articular sprifermin vs placebo on femorotibial joint cartilage thickness in patients with osteoarthritis: the FORWARD randomized clinical trial. JAMA. 2019;322(14):1360-1370. 31593273 10.1001/jama.2019.14735 PMC6784851

[40] Hunt ER Jacobs CA Conley CE-W , et al. Anterior cruciate ligament reconstruction reinitiates an inflammatory and chondrodegenerative process in the knee joint. J Orthop Res. 2021;39(6):1281-1288. 32558951 10.1002/jor.24783

[41] Hunt ER Villasanta-Tezanos AG Butterfield TA Lattermann C Jacobs CA. Upregulation of systemic inflammatory pathways following anterior cruciate ligament injury relates to both cartilage and muscular changes: a pilot study. J Orthop Res. 2020;38(2):387-392. 31517396 10.1002/jor.24467

[42] Ibounig T Simons T Launonen A Paavola M. Glenohumeral osteoarthritis: an overview of etiology and diagnostics. Scand J Surg. 2021;110(3):441-451. 32662351 10.1177/1457496920935018

[43] Ingelsrud LH Granan L-P Terwee CB Engebretsen L Roos EM. Proportion of patients reporting acceptable symptoms or treatment failure and their associated KOOS values at 6 to 24 months after anterior cruciate ligament reconstruction: a study from the Norwegian Knee Ligament Registry. Am J Sports Med. 2015;43(8):1902-1907. 25977523 10.1177/0363546515584041

[44] Ishikawa H Smith KM Wheelwright JC , et al. Rotator cuff muscle imbalance associates with shoulder instability direction. J Shoulder Elbow Surg. 2023;32(1):33-40. 35961497 10.1016/j.jse.2022.06.022

[45] Jacxsens M Elhabian SY Brady SE , et al. Coracoacromial morphology: a contributor to recurrent traumatic anterior glenohumeral instability? J Shoulder Elbow Surg. 2019;28(7):1316-1325.e1. 10.1016/j.jse.2019.01.009 PMC6591074 30928394

[46] Jeong HY Jeon YS Lee DK Rhee YG. Rotator cuff tear with early osteoarthritis: how does it affect clinical outcome after large to massive rotator cuff repair? J Shoulder Elbow Surg. 2019;28(2):237-243. 30337266 10.1016/j.jse.2018.07.022

[47] Jochl OM Afetse EK Garg S , et al. The impact of mental health conditions on clinical and functional outcomes after shoulder arthroplasty: a systematic review. JSES Rev Rep Tech. 2024;4(3):371-378. 39157244 10.1016/j.xrrt.2024.04.014 PMC11329040

[48] Johnson K Zhu S Tremblay MS , et al. A stem cell–based approach to cartilage repair. Science. 2012;336(6082):717-721. 22491093 10.1126/science.1215157

[49] Jung W Lee S Hoon Kim S. The natural course of and risk factors for tear progression in conservatively treated full-thickness rotator cuff tears. J Shoulder Elbow Surg. 2020;29(6):1168-1176. 32044254 10.1016/j.jse.2019.10.027

[50] Kara D Ozcakar L Demirci S Huri G Duzgun I. Blood flow restriction training in patients with rotator cuff tendinopathy: a randomized, assessor-blinded, controlled trial. Clin J Sport Med. 2024;34(1):10. 37706671 10.1097/JSM.0000000000001191

[51] Karimi AH El-Abtah ME Sinkler MA , et al. The role of conservative treatment of glenohumeral joint osteoarthritis: a systematic review. Semin Arthroplasty: JSES. 2024;34(1):34-43.

[52] Kennedy JS Reinke EK Friedman LGM , et al. Protocol for a multicenter, randomised controlled trial of surgeon-directed home therapy vs. outpatient rehabilitation by physical therapists for reverse total shoulder arthroplasty: the SHORT trial. Arch Physiother. 2021;11(1):1-13. 34886910 10.1186/s40945-021-00121-2 PMC8662891

[53] Kircher J Patzer T Magosch P Lichtenberg S Habermeyer P. Osteochondral autologous transplantation for the treatment of full-thickness cartilage defects of the shoulder: results at nine years. J Bone Joint Surg Br. 2009;91(4):499-503. 19336811 10.1302/0301-620X.91B4.21838

[54] Kirschner JS Cheng J Creighton A , et al. Efficacy of ultrasound-guided glenohumeral joint injections of leukocyte-poor platelet-rich plasma versus hyaluronic acid in the treatment of glenohumeral osteoarthritis: a randomized, double-blind controlled trial. Clin J Sport Med. 2022;32(6):558. 35316820 10.1097/JSM.0000000000001029 PMC9481749

[55] Knowles NK Ferreira LM Athwal GS. Premorbid retroversion is significantly greater in type B2 glenoids. J Shoulder Elbow Surg. 2016;25(7):1064-1068. 26895600 10.1016/j.jse.2015.11.002

[56] Kruckeberg BM Leland DP Bernard CD , et al. Incidence of and risk factors for glenohumeral osteoarthritis after anterior shoulder instability: a US population–based study with average 15-year follow-up. Orthop J Sports Med. 2020;8(11):2325967120962515. 10.1177/2325967120962515 PMC7675883 33241059

[57] Lambert B Hedt C Daum J , et al. Blood flow restriction training for the shoulder: a case for proximal benefit. Am J Sports Med. 2021;49(10):2716-2728. 34110960 10.1177/03635465211017524

[58] Lattermann C Conley CE-W Johnson DL , et al. Select biomarkers on the day of anterior cruciate ligament reconstruction predict poor patient-reported outcomes at 2-year follow-up: a pilot study. BioMed Res Int. 2018;2018:1-9. 10.1155/2018/9387809 PMC6076965 30105266

[59] Lattermann C Jacobs CA Proffitt Bunnell M , et al. A multicenter study of early anti-inflammatory treatment in patients with acute anterior cruciate ligament tear. Am J Sports Med. 2017;45(2):325-333. 28146402 10.1177/0363546516666818

[60] Lattermann C Jacobs CA Whale C , et al. Biomarkers on the day of ACL reconstruction and sex predictive of knee-related quality of life at 2-year follow-up. Orthop J Sports Med. 2017;5(3)(suppl 3):2325967117S00127.

[61] Lattermann C Proffitt M Huston LJ , et al. Multicenter Orthopaedic Outcome Network Early Anti-inflammatory Treatment in Patients with Acute ACL Tear” (MOON-AAA) clinical trial. Orthop J Sports Med. 2016;4(7)(suppl 4):2325967116S00189.

[62] Lee J Woodmass JM Bernard CD , et al. Nonoperative management of posterior shoulder instability: what are the long-term clinical outcomes? Clin J Sport Med. 2022;32(2):e116. 10.1097/JSM.0000000000000907 33852434

[63] Li J Wang X Ruan G Zhu Z Ding C. Sprifermin: a recombinant human fibroblast growth factor 18 for the treatment of knee osteoarthritis. Expert Opin Investig Drugs. 2021;30(9):923-930. 10.1080/13543784.2021.1972970 34427483

[64] Lucas J van Doorn P Hegedus E Lewis J van der Windt D. A systematic review of the global prevalence and incidence of shoulder pain. BMC Musculoskelet Disord. 2022;23(1):1073. 36476476 10.1186/s12891-022-05973-8 PMC9730650

[65] Ma GC Bradley KE Jansson H , et al. Surgical complications after reverse total shoulder arthroplasty and total shoulder arthroplasty in the United States. J Am Acad Orthop Surg Glob Res Rev. 2021;5(7):e21.00146. 10.5435/JAAOSGlobal-D-21-00146 PMC8294907 34283038

[66] Mazzocca JL Lowenstein NA Crutchfield CR Collins JE Matzkin EG. Resilience of patients undergoing knee and shoulder arthroscopy procedures. J Am Acad Orthop Surg Glob Res Rev. 2023;7(11):e23.00207. 10.5435/JAAOSGlobal-D-23-00207 PMC10653576 37967061

[67] Metzger CM Farooq H Merrell GA , et al. Efficacy of a single, image-guided corticosteroid injection for glenohumeral arthritis. J Shoulder Elbow Surg. 2021;30(5):1128-1134. 32858193 10.1016/j.jse.2020.08.008

[68] Mihata T Lee Y McGarry MH Abe M Lee TQ. Excessive humeral external rotation results in increased shoulder laxity. Am J Sports Med. 2004;32(5):1278-1285. 15262654 10.1177/0363546503262188

[69] Millett PJ Horan MP Pennock AT Rios D. Comprehensive Arthroscopic Management (CAM) procedure: clinical results of a joint-preserving arthroscopic treatment for young, active patients with advanced shoulder osteoarthritis. Arthroscopy. 2013;29(3):440-448. 23544687 10.1016/j.arthro.2012.10.028

[70] Misir A Uzun E Kizkapan TB , et al. Factors associated with the development of early- to mid-term cuff-tear arthropathy following arthroscopic rotator cuff repair. J Shoulder Elbow Surg. 2021;30(7):1572-1580. 33038498 10.1016/j.jse.2020.09.016

[71] Moroder P Siegert P Coifman I , et al. Scapulothoracic orientation has a significant influence on the clinical outcome after reverse total shoulder arthroplasty. J Shoulder Elbow Surg. 2024;33(10):2159-2170. 38537767 10.1016/j.jse.2024.02.018

[72] Moverman MA Puzzitiello RN Menendez ME , et al. Rotator cuff fatty infiltration and muscle atrophy: relation to glenoid deformity in primary glenohumeral osteoarthritis. J Shoulder Elbow Surg. 2022;31(2):286-293. 34390840 10.1016/j.jse.2021.07.007

[73] Neer CS 2nd Craig EV Fukuda H. Cuff-tear arthropathy. J Bone Joint Surg. 1983;65(9):1232. 6654936

[74] Nové-Josserand L Nerot C Colotte P , et al. Reverse shoulder arthroplasty for primary glenohumeral osteoarthritis: significantly different characteristics and outcomes in shoulders with intact vs. torn rotator cuff. J Shoulder Elbow Surg. 2024;33(4):850-862. 37633591 10.1016/j.jse.2023.07.027

[75] O’Brien J Grebenyuk J Leith J Forster BB. Frequency of glenoid chondral lesions on MR arthrography in patients with anterior shoulder instability. Eur J Radiol. 2012;81(11):3461-3465. 22698712 10.1016/j.ejrad.2012.05.013

[76] Ogawa K Yoshida A Matsumoto H Takeda T. Outcome of the open Bankart procedure for shoulder instability and development of osteoarthritis: a 5- to 20-year follow-up study. Am J Sports Med. 2010;38(8):1549-1557. 20505055 10.1177/0363546510363464

[77] O’Neill DC Christensen GV Hillyard B , et al. Glenoid retroversion associates with deltoid muscle asymmetry in Walch B-type glenohumeral osteoarthritis. JSES Int. 2021;5(2):282-287. 33681850 10.1016/j.jseint.2020.10.012 PMC7910726

[78] Oswald A Menze J Hess H , et al. Effect of patient-specific scapular morphology on the glenohumeral joint force and shoulder muscle force equilibrium: a study of rotator cuff tear and osteoarthritis patients. Front Bioeng Biotechnol. 2024;12:1355723. 38807649 10.3389/fbioe.2024.1355723 PMC11132099

[79] Ozbaydar M Elhassan B Diller D , et al. Results of arthroscopic capsulolabral repair: Bankart lesion versus anterior labroligamentous periosteal sleeve avulsion lesion. Arthroscopy. 2008;24(11):1277-1283. 18971059 10.1016/j.arthro.2008.01.017

[80] Parada SA Flurin P-H Wright TW , et al. Comparison of complication types and rates associated with anatomic and reverse total shoulder arthroplasty. J Shoulder Elbow Surg. 2021;30(4):811-818. 32763380 10.1016/j.jse.2020.07.028

[81] Pogorzelski J Fritz EM Horan MP , et al. Failure following arthroscopic Bankart repair for traumatic anteroinferior instability of the shoulder: is a glenoid labral articular disruption (GLAD) lesion a risk factor for recurrent instability? J Shoulder Elbow Surg. 2018;27(8):e235-e242. 10.1016/j.jse.2018.02.055 29730139

[82] Porcellini G Campi F Pegreffi F Castagna A Paladini P. Predisposing factors for recurrent shoulder dislocation after arthroscopic treatment. J Bone Joint Surg. 2009;91(11):2537. 19884424 10.2106/JBJS.H.01126

[83] Pougès C Hardy A Vervoort T , et al. Arthroscopic Bankart repair versus immobilization for first episode of anterior shoulder dislocation before the age of 25: a randomized controlled trial. Am J Sports Med. 2021;49(5):1166-1174. 33705240 10.1177/0363546521996381

[84] Razmjou H . Cuff tear arthropathy. In: Razmjou H Christakis M , eds. Clinical and Radiological Examination of the Shoulder Joint: A Guide for Advanced Practice Physiotherapists. Springer International Publishing; 2022:59-74. Accessed September 8, 2024. 10.1007/978-3-031-10470-1_5

[85] Reineck JR Krishnan SG Burkhead WZ. Early glenohumeral arthritis in the competing athlete. Clin Sports Med. 2008;27(4):803-819. 19064157 10.1016/j.csm.2008.07.004

[86] Riff AJ Yanke AB Shin JJ Romeo AA Cole BJ. Midterm results of osteochondral allograft transplantation to the humeral head. J Shoulder Elbow Surg. 2017;26(7):e207-e215. 10.1016/j.jse.2016.11.053 28162881

[87] Ruckstuhl H de Bruin ED Stussi E Vanwanseele B. Post-traumatic glenohumeral cartilage lesions: a systematic review. BMC Musculoskelet Disord. 2008;9(1):107. 18651982 10.1186/1471-2474-9-107 PMC2503981

[88] Rugg CM Gallo RA Craig EV Feeley BT. The pathogenesis and management of cuff tear arthropathy. J Shoulder Elbow Surg. 2018;27(12):2271-2283. 30268586 10.1016/j.jse.2018.07.020

[89] Scanaliato JP Sandler AB Baird MD , et al. Glenoid microfracture in active-duty military patients: minimum 5-year follow-up demonstrates 75% survival. JSES Int. 2023;7(1):86-92. 36820416 10.1016/j.jseint.2022.09.011 PMC9937841

[90] Schmitt H Hansmann HJ Brocai DRC Loew M. Long term changes of the throwing arm of former elite javelin throwers. Int J Sports Med. 2001;22:275-279. 11414670 10.1055/s-2001-13814

[91] Schneider DJ Tibone JE McGarry MH , et al. Biomechanical evaluation after five and ten millimeter anterior glenohumeral capsulorrhaphy using a novel shoulder model of increased laxity. J Shoulder Elbow Surg. 2005;14(3):318-323. 15889033 10.1016/j.jse.2004.07.006

[92] Schoell K Crabb R Simpson E , et al. Preoperative corticosteroid injections are associated with a higher periprosthetic infection rate following primary total shoulder arthroplasty: a systematic review and meta-analysis. J Shoulder Elbow Surg. 2024;33(12):2734-2742. 39002882 10.1016/j.jse.2024.05.025

[93] Schoenfeldt TL Trenhaile S Olson R. Glenohumeral osteoarthritis: frequency of underlying diagnoses and the role of arm dominance—a retrospective analysis in a community-based musculoskeletal practice. Rheumatol Int. 2018;38(6):1023-1029. 29423534 10.1007/s00296-018-3989-1

[94] Spiegl UJ Horan MP Smith SW Ho CP Millett PJ. The critical shoulder angle is associated with rotator cuff tears and shoulder osteoarthritis and is better assessed with radiographs over MRI. Knee Surg Sports Traumatol Arthrosc. 2016;24(7):2244-2251. 25820655 10.1007/s00167-015-3587-7

[95] Thomeé R. A comprehensive treatment approach for patellofemoral pain syndrome in young women. Phys Ther. 1997;77(12):1690-1703. 9413448 10.1093/ptj/77.12.1690

[96] Till SE Reinholz AK Lee J , et al. Patient age and surgical intervention as risk factors for the development of osteoarthritis after posterior shoulder instability: a population-based study. Orthop J Sports Med. 2022;10(7):23259671221112973. 10.1177/23259671221112973 PMC9344110 35928179

[97] Tokish JM Kissenberth MJ Tolan SJ , et al. Resilience correlates with outcomes after total shoulder arthroplasty. J Shoulder Elbow Surg. 2017;26(5):752-756. 28190668 10.1016/j.jse.2016.12.070

[98] Visotsky JL Basamania C Seebauer L Rockwood CA Jensen KL. Cuff tear arthropathy: pathogenesis, classification, and algorithm for treatment. JBJS. 2004;86(suppl 2):35. 15691107

[99] Walch G Badet R Boulahia A Khoury A. Morphologic study of the glenoid in primary glenohumeral osteoarthritis. J Arthroplasty. 1999;14(6):756-760. 10512449 10.1016/s0883-5403(99)90232-2

[100] Walch G Boileau P Noel E Donell ST. Impingement of the deep surface of the supraspinatus tendon on the posterosuperior glenoid rim: an arthroscopic study. J Shoulder Elbow Surg. 1992;1(5):238-245. 22959196 10.1016/S1058-2746(09)80065-7

[101] Wall KC Politzer CS Chahla J Garrigues GE. Obesity is associated with an increased prevalence of glenohumeral osteoarthritis and arthroplasty: a cohort study. Orthop Clin. 2020;51(2):259-264. 10.1016/j.ocl.2019.12.001 32138863

[102] Wang KC Frank RM Cotter EJ , et al. Long-term clinical outcomes after microfracture of the glenohumeral joint: average 10-year follow-up. Am J Sports Med. 2018;46(4):786-794. 29373801 10.1177/0363546517750627

[103] Wang Z Liu X Jiang K , et al. Intramuscular brown fat activation decreases muscle atrophy and fatty infiltration and improves gait after delayed rotator cuff repair in mice. Am J Sports Med. 2020;48(7):1590-1600. 32282238 10.1177/0363546520910421

[104] Weinstein DM Bucchieri JS Pollock RG Flatow EL Bigliani LU. Arthroscopic debridement of the shoulder for osteoarthritis. Arthroscopy. 2000;16(5):471-476. 10882441 10.1053/jars.2000.5042

[105] Wermers J Schliemann B Raschke MJ , et al. The glenolabral articular disruption lesion is a biomechanical risk factor for recurrent shoulder instability. Arthrosc Sports Med Rehabil. 2021;3(6):e1803-e1810. 10.1016/j.asmr.2021.08.007 PMC8689271 34977634

[106] Werthel J-D Dufrenot M Schoch BS , et al. Are glenoid retroversion, humeral subluxation, and Walch classification associated with a muscle imbalance? J Shoulder Elbow Surg. 2024;33(7):1493-1502. 38242526 10.1016/j.jse.2023.11.027

[107] Wolfe JA Elsenbeck M Nappo K , et al. Effect of posterior glenoid bone loss and retroversion on arthroscopic posterior glenohumeral stabilization. Am J Sports Med. 2020;48(11):2621-2627. 32813547 10.1177/0363546520946101

[108] Yazici Y McAlindon TE Gibofsky A , et al. Lorecivivint, a novel intraarticular CDC-like kinase 2 and dual-specificity tyrosine phosphorylation-regulated kinase 1A inhibitor and Wnt pathway modulator for the treatment of knee osteoarthritis: a phase II randomized trial. Arthritis Rheumatol. 2020;72(10):1694-1706. 32432388 10.1002/art.41315 PMC7589351

[109] Zaid MB Young NM Pedoia V , et al. Anatomic shoulder parameters and their relationship to the presence of degenerative rotator cuff tears and glenohumeral osteoarthritis: a systematic review and meta-analysis. J Shoulder Elbow Surg. 2019;28(12):2457-2466. 31353303 10.1016/j.jse.2019.05.008

[110] Zhang Y Chen H Wu J , et al. Deficiency of Cbfβ in articular cartilage leads to osteoarthritis-like phenotype through Hippo/Yap, TGFβ, and Wnt/β-catenin signaling pathways. Int J Biol Sci. 2024;20(6):1965-1977. 38617544 10.7150/ijbs.90250 PMC11008268

[111] Zhou K Li Y-J Soderblom EJ , et al. A “best-in-class” systemic biomarker predictor of clinically relevant knee osteoarthritis structural and pain progression. Sci Adv. 2023;9(4):eABQ5095. 10.1126/sciadv.abq5095 PMC9876540 36696492

